# Effect of Alkali Treatment on the Mechanical Properties of Anion-Exchange Membranes with a Poly(vinyl Chloride) Backing and Binder

**DOI:** 10.3390/membranes10110344

**Published:** 2020-11-16

**Authors:** Shoichi Doi, Ikuo Taniguchi, Masahiro Yasukawa, Yuriko Kakihana, Mitsuru Higa

**Affiliations:** 1 Astom Corporation, 1-1 Mikagecho, Syunan, Yamaguchi 745-8648, Japan; s.doi@astom-corp.jp; 2 Graduate School of Sciences and Technology for Innovation, Yamaguchi University, 2-16-1 Tokiwadai, Ube, Yamaguchi 755-8611, Japan; masahiro_yasukawa@toyobo.jp (M.Y.); kakihana@yamaguchi-u.ac.jp (Y.K.); 3 International Institute for Carbon-Neutral Energy Research (WPI-I2CNER), Kyushu University, 744 Motooka, Nishi-ku, Fukuoka 819-0395, Japan; ikuot@i2cner.kyushu-u.ac.jp; 4 Blue Energy Center for SGE Technology (BEST), 2-16-1 Tokiwadai, Ube, Yamaguchi 755-8611, Japan

**Keywords:** anion-exchange membrane, degradation, alkali, polyvinyl chloride, cleaning in place, dehydrochlorination, mechanical properties

## Abstract

An alkali treatment under various operating conditions is conducted on a commercial anion-exchange membrane containing poly(vinyl chloride) (PVC) as a backing and binder to study the effect of the treatment on the mechanical properties by both Müllen burst and tensile tests. Contrary to our expectations, the Müllen burst pressure and tensile strain at break improved significantly after the alkali treatment in comparison to the pristine membrane and then decreased as the treatment period progressed. A good correlation is observed between the area below the stress–strain curve and burst pressure. To understand the obtained results, the PVC degradates are recovered by Soxhlet extraction and characterized via nuclear magnetic resonance and gel permeation chromatography. It is discovered that the PVC main chains degraded in the alkali solution. We propose a composite model to explain the burst pressure improvement mechanism by the change in the chemical structure of the PVC binder.

## 1. Introduction

In industrial products, not only good performances and cost effectiveness, but also durability must be guaranteed over a long service duration. Electrodialysis (ED) using an ion-exchange membrane (IEM) is no exception, and long-term stability in both electrochemical and mechanical properties is crucial for the development of IEMs. ED process suppliers are required to propose better operating methods for customers. It is crucial to identify and mitigate issues as they arise.

In commercial ED plants, the deterioration in the electrochemical properties of IEMs can be identified based on changes in the operating voltage, electric current, and production amount of the target products. By contrast, the mechanical properties, another important factor in determining the lifetime of IEMs, are difficult to analyze until a fatal event occurs, such as breakage.

In particular, anion-exchange membranes (AEMs) are often contaminated by the substances to be treated and other impurities, which often cause problems such as an increased operation voltage. Hence, chemicals such as alkalis are often used to decompose and remove contaminants to restore the performance without disassembling the ED stack and cleaning in place (CIP). We have previously focused on the effect of alkali contact on the electrochemical characteristics of AEMs [1]. 

In addition, the effect of alkali contact on the mechanical characteristics of AEMs has been discussed [2,3,4,5,6]. Sata reported that the Müllen burst pressure of custom-developed chloromethylated polysulfone AEMs decreased to less than 1/3 that of the original AEMs after treatment in an alkali solution [2]. Dammak et al. systematically studied changes in the mechanical strength of IEMs by alkali treatment [3,4,5,6]. Ghalloussi et al. investigated the mechanical properties of AEMs and cation-exchange membranes (CEMs) after 2 years of operation in a commercial food plant. They discovered a 4% and a 63% decrease in the Young’s modulus and rupture strength for the AEMs, respectively, compared with the pristine membranes, whereas 6% and 13% for the CEMs [3]. Although both membranes had a woven PVC backing, scanning electron microscopy (SEM) observations showed that they were peeled from the backing with degradation of the sulfonated polystyrene [4]. Vasquez et al. focused on AEMs and investigated the deterioration of the membranes until the “lifetime of the membranes” after desalting whey for several thousand hours on an ED commercial plant. The Young’s modulus was down to 80% that of the pristine material, whereas the breaking elongation decreased to 42%. The area below the strain–stress (SS) curve decreased by 24% compared with the original value. The membrane features changed from rigid and tough to rigid and brittle upon alkali treatment [5]. Furthermore, Vasquez et al. immersed a homogeneous AEM and CEM at room temperature in 2 M NaOH and HCl for up to 700 h. In addition, a cleaning cycle was performed for the AEM and CEM up to 400 times at 40 °C, as follows: 0.1 M NaOH for 30 min, a water wash, 0.1 M HCl for 30 min, and a water wash. They also investigated the effect of the cleaning cycle on the mechanical performance [6]. However, no studies have systematically investigated the effects of alkali treatment conditions (temperature, concentration, and time) on the mechanical properties of IEMs.

In this study, we conducted an evaluation focusing on the mechanical properties such as burst pressure and tensile strength, which are particularly useful in the field, and clarified the quantitative change in the mechanical strength of AEMs before and after alkali treatment. Furthermore, we analyzed their chemical structure, considered the mechanism of chemical structure change, and analyzed their contribution to the change in the mechanical strength of AEMs.

## 2. Materials and Methods 

### 2.1. Experimental Materials

A commercially available anion-exchange membrane, Neosepta^®^ AMX (Astom Corporation, Tokyo, Japan), was used in this study. AMX is a standard-grade membrane containing PVC in the backing and binder.

### 2.2. Alkali Treatment

A systematic alkali treatment was performed. The temperature was set to 40 °C, 60 °C, and 80 °C; the NaOH concentrations were 0.01 M, 0.1 M, and 1 M; and the treatment times were 3, 24, and 168 h. For details regarding the alkali treatment, please refer to a previous paper [1].

### 2.3. Physical Property Evaluation

#### 2.3.1. Burst Pressure 

**Figure 1:**
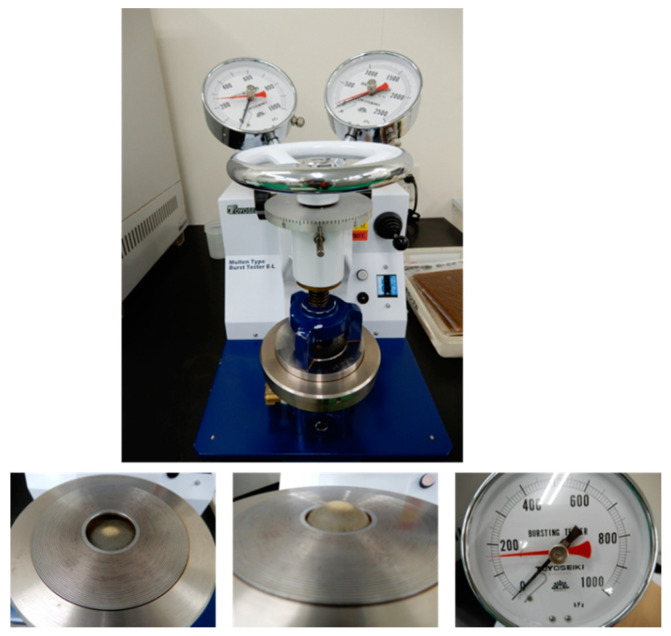
A Müllen burst pressure measuring device: (**top**) overall; (**left**) before test; (**center**) during test; (**right**) red indicator needle.

In the ED industry, the Müllen burst pressure is a representative property to quantify the mechanical strength owing to its simple operation. The top of Figure 1 shows a photograph of the Müllen burst pressure measuring device (Toyo Seiki Seisakusho, Tokyo, Japan). The burst pressure was measured as follows: After the alkali treatment, the AEMs were immersed in 0.5N NaCl to equilibrate to the Cl type. The membrane sample was sandwiched and fixed by the upper and lower pressing plates with an opening of diameter ~3 cm (Figure 1, left). Stress was applied by the expansion of the rubber under the internal pressure from the lower side of the opening (Figure 1, center), and the pressure at the moment of bursting was read, based on the red indicator needle (Figure 1, right). The units was MPa. The normalized burst pressure (%) was calculated using Equation (1).

Normalized burst pressure (%) = Burst pressure (MPa) of test piece that passed treatment test/Burst pressure (MPa) of pristine membrane that failed treatment test(1)

#### 2.3.2. Tensile Strength 

Compared with the Müllen burst pressure measurement, the tensile test provides more information through measurements. Therefore, a tensile test was performed to analyze the mechanical characteristics.

The AMX membrane exhibits anisotropy with different strength characteristics in the longitudinal direction (MD: machine direction) and width direction (TD: traverse direction), owing to the characteristics of the woven backing fabric. In this study, only the MD was measured.

A tensile test sample was cut from the alkali treatment sample that was well equilibrated with 0.5 N NaCl. The test specimen measured were 10 mm in width, 50 mm in length, and 20 mm in effective length. Tensile tests were conducted at a displacement rate of 50 mm/min to determine the tensile properties at room temperature. The thickness of each specimen was measured. A tensile tester “Tensilon” (manufactured by A & D, Tokyo, Japan) was used to perform the slack correction in a wet state. The stress and strain at break were calculated using Equations (2) and (3).

Stress at break (N/mm^2^) = Load at break/Sample thickness/Sample width (2)

Strain at break (%) = Elongation at break/Effective sample length(3)

Young’s modulus (N/mm^2^) = Slope of initial proportional section of stress–strain curve (4)

To discuss the changes in various physical properties after the alkali treatment, the stress, strain, area below the SS curve, and Young’s modulus of each membrane were normalized by the stress at break, strain at break, area below the SS curve to break, and the Young’s modulus of the pristine membrane.

### 2.4. Chemical Structure Analysis

To investigate the effect of the alkali treatment, changes in the chemical structure of the PVC and the mechanical properties of the treated AEMs were investigated. The AEMs were immersed in 1 M NaOH at 80 °C for 5 min, 10 min, 30 min, 3 h, and 24 h. The PVC degradation of the AEMs by the treatment was extracted in tetrahydrofuran (THF) via Soxhlet extraction. In brief, the AEMs were placed in a thimble filter (Advantec, Tokyo, Japan) and a THF-soluble fraction was extracted at 85–90 °C for 48–58 h. The resulting THF solution was condensed using a rotary evaporator and added to hexane. The precipitate (Extract A) was recovered by filtration, whereas a hexane-soluble part (extract B) was obtained after the evaporation of the filtrate. The AEMs after extraction were termed as “Residue C.”

With Extract A, the molecular weights and polydispersity were determined via gel permeation chromatography (GPC) on a JASCO (JASCO, Tokyo, Japan) LC system with an RI-2013Plus RI detector. A Shodex GPC LF-804 column (lot no. E2370051, Showa Denko, Tokyo, Japan) was used to fractionate the samples, and THF was used as the eluent at a flow rate of 0.5 mL/min at 40 °C. The chemical structures of the extracts were analyzed via ^1^H and ^13^C NMR on a Bruker ADVANCE III (Bruker, Yokohama, Japan), operating at 600 and 150 MHz, respectively. The NMR spectra were recorded in deuterated THF at 25 °C. 

The mechanical properties of the treated AEMs and residue C were measured using an EZ-SX (Shimadzu, Kyoto, Japan) with a 500 N load cell. The test specimen measured 5 mm in width, 30 mm in length, and 12 mm in effective length. Tensile tests were conducted at a displacement rate of 1 mm/min to determine the tensile properties at room temperature (*n* = 5). The test conditions differed from those used in the tensile test described in Section 2.3.2.

## 3. Results and Discussion

### 3.1. Burst Pressure

**Figure 2:**
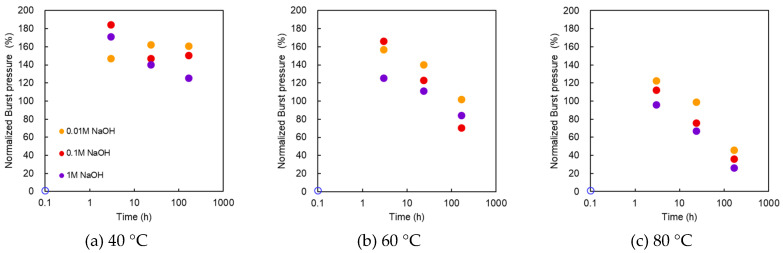
Normalized burst pressure as a function of treatment time at various NaOH concentrations and temperatures. Temperature of the treatment solutions: (**a**) 40 °C; (**b**) 60 °C; (**c**) 80 °C.

The Müllen burst test was performed to determine the normalized burst pressure. Figure 2 shows the normalized burst pressure as a function of treatment time at various NaOH concentrations and temperatures. The horizontal axis is plotted in logarithmic time. Regarding the electrochemical characteristics, when the AMX membrane was immersed in alkali at 40 °C for approximately 1 week, the water content increased, the electrical resistance decreased, and the ion selective permeability decreased [1]. 

However, it is noteworthy that the burst pressure increased once and then decreased after 1 week of treatment. To the authors’ best knowledge, studies regarding the strength of AEMs being improved by alkali contact have not been reported.

In the alkali treatment at 40 °C, the maximum burst pressure was recorded at 24 h and 0.01 M, whereas the maximum was recorded at 0.1 M and 1 M at 3 h. These results suggest that under a higher NaOH concentration or temperature, the maximum burst pressure occurred at a shorter treatment time. At 0.1 M, the strength was almost double that of the pristine at 3 h and 150% at 168 h. The case of 0.1 M NaOH and 168 h was equivalent to 3 years of standard CIP conditions (1 h treatment with 0.1 M NaOH once a week).

In the treatment at 60 °C, the maximum value was recorded at 3 h, even at 0.01 M, and at 1 M, it was lower than the initial value after approximately 40 h.

At 80 °C, the strength increased from the initial value at treatment concentrations of 0.01 and 0.1 M for 3 h, but the strength of the samples for all the treatment concentrations decreased from the initial value after approximately 20 h. 

When a membrane is subjected to the Müllen burst test, it is assumed that the tensile stress is generated in a two-dimensional direction on the membrane surface. Therefore, the increase in burst pressure can be associated with the increase in the tensile factors. 

To clarify the reason for the burst pressure increase, a tensile test was performed. 

### 3.2. Tensile Test

**Figure 3:**
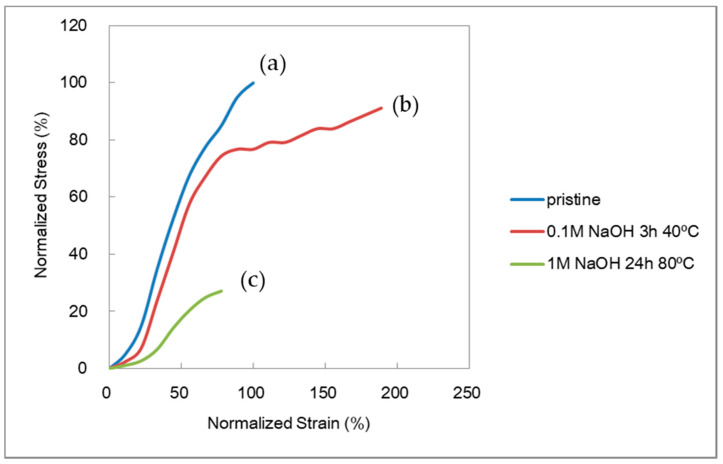
Typical normalized stress–strain (SS) curves of the AMX membrane: (**a**) pristine (blue); (**b**) membrane mildly treated in 0.1 M NaOH for 3 h at 40 °C (red); (**c**) membrane severely treated in 1 M 24 h at 80 °C (green).

Figure 3 shows typical normalized SS curves for the AMX membrane: (a) a pristine membrane; (b) a membrane treated in 0.1 M NaOH for 3 h at 40 °C; and (c) a membrane treated in 1 M for 168 h at 80 °C. The pristine membrane showed little elongation before being fractured. In (b), a similar SS curve was observed before a yield point to the pristine membrane, but the membrane showed a higher strain. The higher strain might have contributed to the higher burst pressure. In (c), the AMX membrane indicated a much lower stress and strain. 

**Figure 4:**
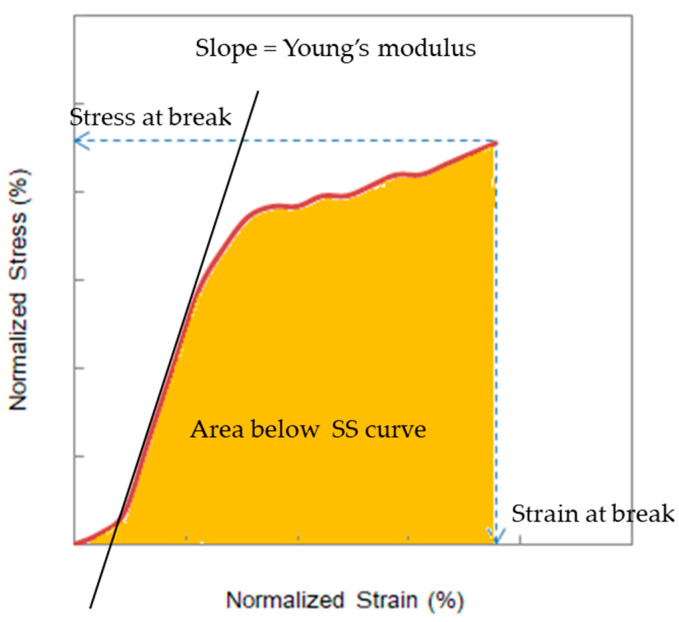
Four factors obtained from the SS curve.

As shown in Figure 4, four factors were obtained from each SS curve, i.e., stress at break, strain at break, the Young’s modulus, and the area below the SS curve. Vasquez et al. investigated the area below the SS curve in the analysis of deteriorated AEMs [4]. To determine the key factor that dominates the burst pressure, the four factors were plotted as a function of treatment time at various NaOH concentrations and temperatures; it was observed that the plots showed a similar trend to that of the burst pressure.

#### 3.2.1. Stress at Break

**Figure 5:**
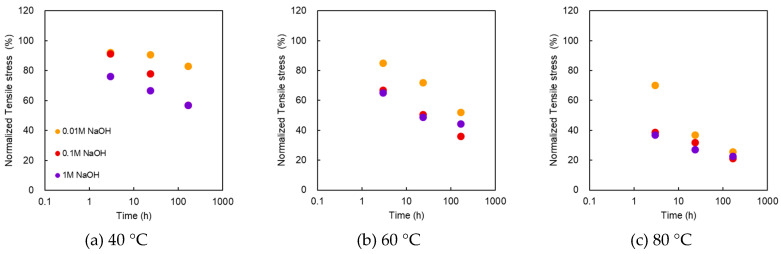
Normalized stress at break as a function of treatment time at various NaOH concentrations and temperatures. Temperatures of the treatment solutions: (**a**) 40 °C; (**b**) 60 °C; (**c**) 80 °C.

Figure 5 shows the normalized stress at break as a function of treatment time at various NaOH concentrations and temperatures. As shown, the maximum point stress decreased monotonically as the alkali treatment time progressed. For 0.01, 0.1, and 1 M at 40 °C, the samples immersed in higher concentrations of alkali showed a lower stress at break. At 60 °C, the stress at break was lower than 40 °C, and it did not differ significantly between 0.1 and 1 M. However, it decreased further at 80 °C, and after 168 h, it remained almost the same for all concentrations. The plot trend of the stress at break differed from that of the burst pressure.

#### 3.2.2. Strain at Break

**Figure 6:**
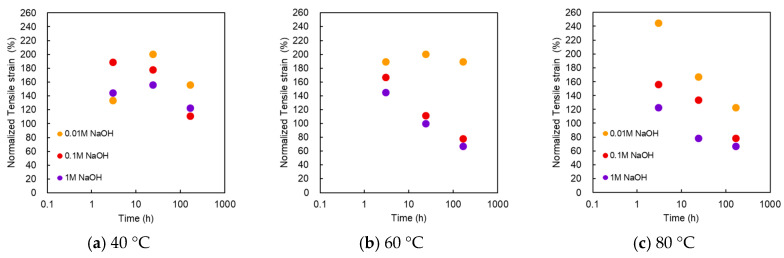
Normalized strain at break as a function of treatment time at various NaOH concentrations and temperatures. Temperatures of the treatment solutions: (**a**) 40 °C; (**b**) 60 °C; (**c**) 80 °C.

Figure 6 shows the normalized strain at break as a function of treatment time at various NaOH concentrations and temperatures. Interestingly, the strain at break showed a significant increase. In the alkali treatment with 0.01 M NaOH at 40 °C, the strain at break increased with the treatment time, reaching a maximum value at 24 h, and then decreased. The maximum strain was almost twice the initial value. The maximum value was recorded at 3 h in case of 0.1 M NaOH. All points within 168 h exceeded the initial value. At 60 °C, the maximum strain at break appeared at 0.01 M for 24 h, but it decreased monotonically and decreased below the initial value for 0.1 and 1 M. At 80 °C, it decreased monotonically after 3 h. The plots of 1 M at 3 h, 0.1 M at 24 h, and 0.01 M at 168 h indicated values that were higher than the initial value. This trend differed from that of the burst pressure.

#### 3.2.3. Young’s Modulus

**Figure 7:**
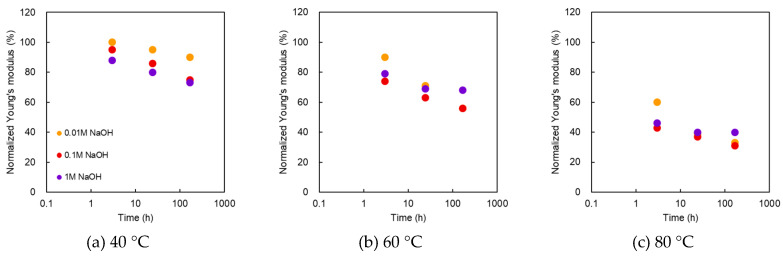
Normalized Young’s modulus as a function of treatment time at various NaOH concentrations and temperatures. Temperatures of the treatment solutions: (**a**) 40 °C; (**b**) 60 °C; (**c**) 80 °C.

Figure 7 shows the normalized Young’s modulus as a function of treatment time at various NaOH concentrations and temperatures. After the alkali treatment, the Young’s modulus decreased monotonically with time at all temperatures but decreased as the temperature increased. At 40 °C, the Young’s modulus for 0.01, 0.1, and 1 M decreased as the concentration increased; however, at 60 °C and 80 °C, no difference was observed for all the concentrations. The plot trend of the Young’s modulus differed from that of the burst pressure.

#### 3.2.4. Area below the SS Curve

**Figure 8:**
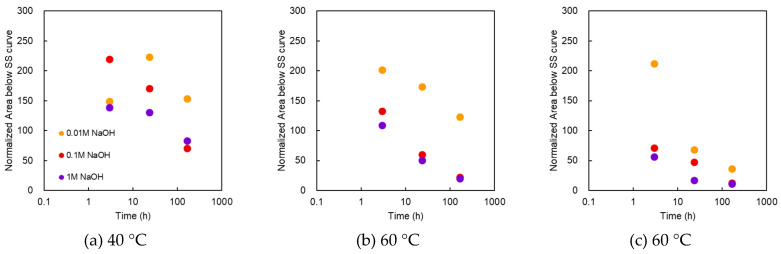
Normalized area below the SS curve as a function of treatment time at various NaOH concentrations and temperatures. Temperatures of the treatment solutions: (**a**) 40 °C; (**b**) 60 °C; (**c**) 80 °C.

Figure 8 shows the area below the SS curve as a function of treatment time at various NaOH concentrations and temperatures. At 40 °C, the maximum value was recorded at 24 h for 0.01 M, whereas the maximum value was recorded at 3 h a for 0.1 and 1 M. At 0.1 M for 3 h, the area was almost double that of the pristine membrane. When immersed for 168 h, the value became slightly lower than the initial value. At 60 °C and 0.01 M, the maximum value was recorded at 3 h. In the case of 1 M, it decreased below the initial value after a few hours. At 80 °C, the area below the SS curve increased from the initial value for 0.01 M at 3 h, but the other samples indicated values below the initial value. At the same treatment time, the area decreased as the alkali concentration increased. The overall behavior exhibited was similar to that of the burst pressure.

**Figure 9:**
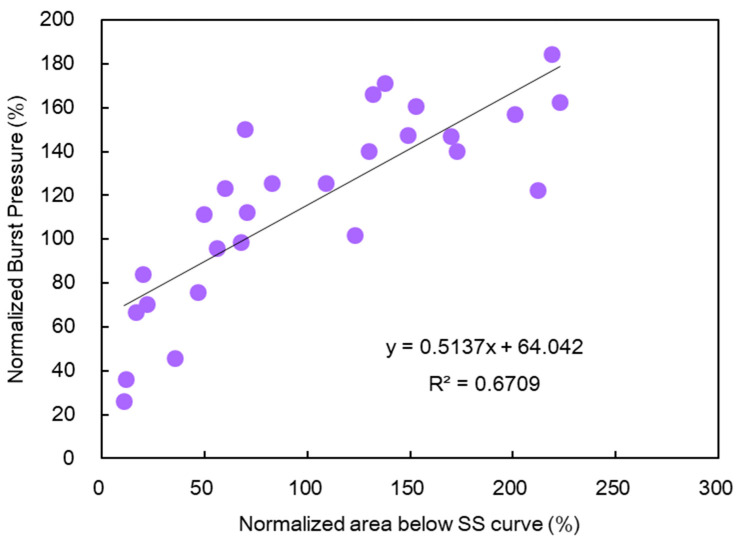
Correlation between the burst pressure and the area below the SS curve.

The area below the SS curve in Figure 8 was almost the same as that of the burst pressure in Figure 2, despite some numerical differences. Therefore, as in Figure 9, the correlation between the normalized area below the SS curve on the horizontal axis and the normalized burst pressure on the vertical axis was examined. The correlation coefficient (R^2^) was 0.6709, which suggested a correlation, although weak. Meanwhile, when the normalized strain at break was represented in the vertical axis, the correlation coefficient (R^2^) decreased to 0.4121. The area below the SS curve exhibited a higher correlation coefficient with the burst pressure.

Because the horizontal axis of the SS curve denoted strain and the vertical axis denoted stress, the area below the SS curve was assumed to represent the amount of work or energy until burst of a membrane. 

A systematic alkali attack test on the AEM helped to confirm the following. The standard CIP condition, as recommended by the supplier, is that an AEM and 0.1 M NaOH solution should be in contact for 1 h at 40 °C. If this process is realized 52 times per year for 3 years, the cumulative immersion time will be 156 h. The equivalent load observed at the 168th hour of the immersion test conducted in this study almost corresponded to the standard CIP. At 168 h, the burst strength and area below the SS curve are higher than those observed for the pristine membrane. Hence, no problem is expected if CIP is performed solely by passing the above conditioned solution through the ED stack. 

However, the AEM swells during the alkaline treatment and shrinks during the neutralization treatment. This dimensional change facilitates the deterioration of the AEM [6]. In addition to the immersion or passing of the solution, in practical maintenance scenarios, operations such as physical rubbing to remove fouling substances may be required. If such additional operations are performed, the deterioration of the AEM will inevitably progress.

### 3.3. Chemical Structure Analysis

**Figure 10:**
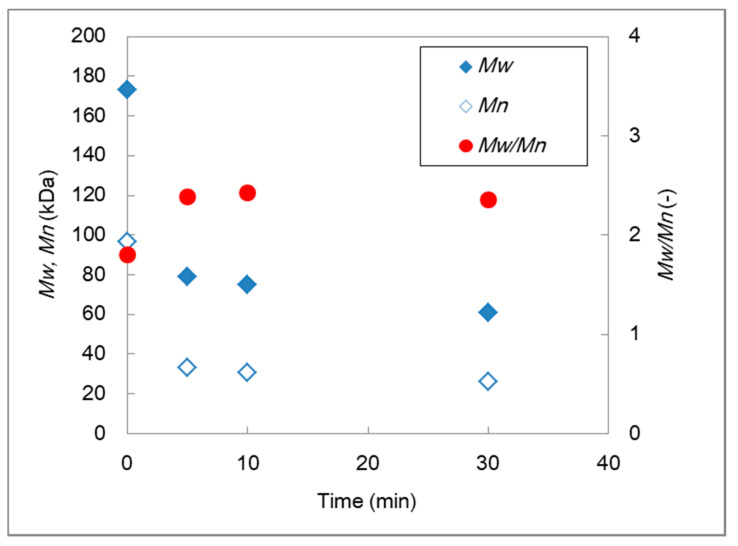
Changes in the molecular weights and dispersity of PVC by alkali treatment.

The deterioration in the mechanical properties of the AEMs is attributable to the degradation of the PVC skeleton. The PVC was extracted in THF via Soxhlet extraction, whereas changes in the molecular weight and the polydispersity of the polymeric fraction (Extract A) were determined via GPC, as shown in Figure 10. Both the number-average molecular weight (*M*_n_) and weight-average molecular weight (*M*_w_) decreased as the alkali treatment time progressed. Meanwhile, the polydispersity (*M*_w_/*M*_n_) increased with the treatment time. The obtained results suggest the random scission of the PVC chain under alkali treatment conditions. Rapid PVC degradation appeared to have occurred in the first 5 min, from 175 to 80 kDa in *M*_w_; thereafter, the molecular weight decreased less with a constant polydispersity. This might be because only a hexane-insoluble polymeric fraction was collected by the extraction (Extract A), whereas the significantly lower molecular weight PVC in Extract B was outside of the analysis targets. However, the GPC results suggested that the PVC degradation in such high pH conditions resulted primarily in a change in the mechanical properties of the AEMs.

**Figure 11:**
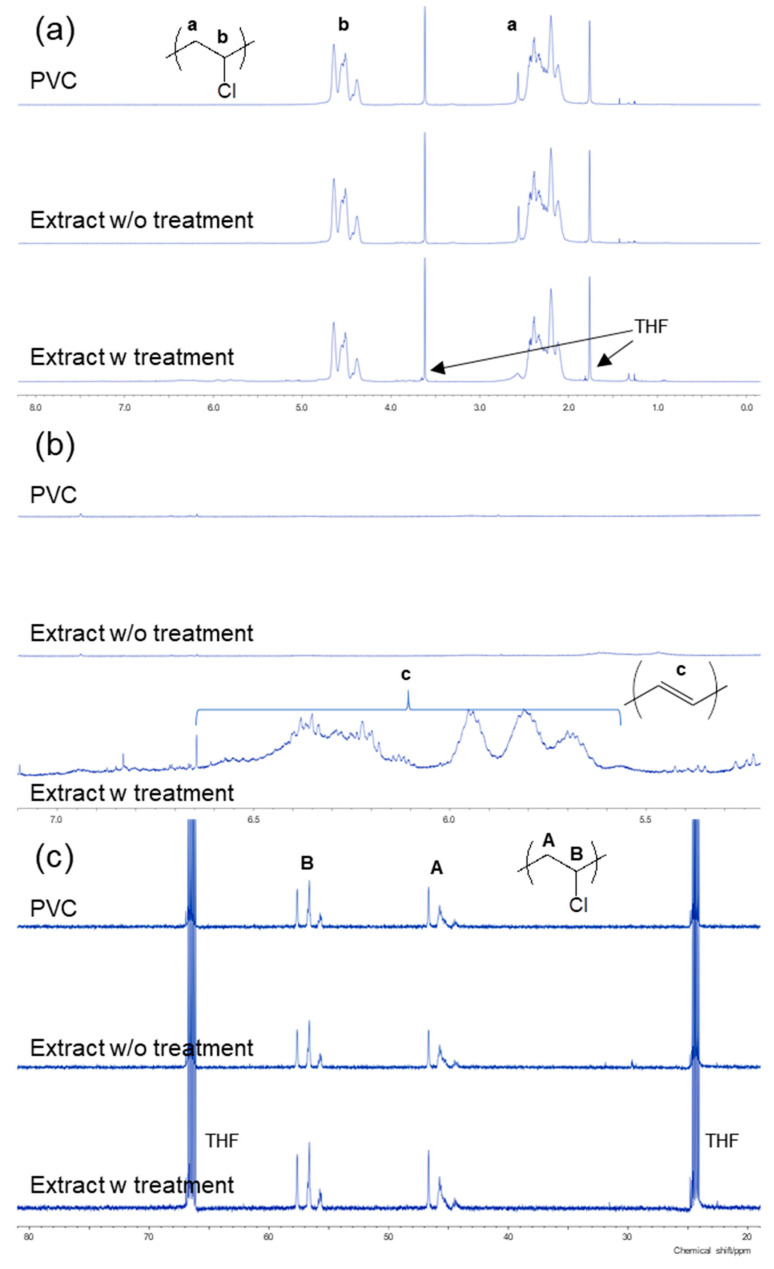
(**a**,**b**) ^1^H NMR and (**c**) ^13^C NMR spectra of PVC, an extract from the AMX membrane without alkali treatment, and an extract from the AMX membrane after alkali treatment for 30 min in 1 M NaOH at 80 °C. Panel (**b**) is an enlarged view of the polyene region of (**a**).

The PVC degradation owing to the alkali treatment was investigated via ^1^H and ^13^C NMR. Figure 11a shows the ^1^H NMR spectra of pristine PVC in an IEM, an extract from the AMX membrane without alkali treatment, and an extract from the AMX membrane after alkali treatment for 30 min in 1 M NaOH at 80 °C. In all cases, two significant proton peaks were observed at 2.1–2.6 and 4.3–4.7 ppm, which were assigned to methylene (CH_2_) and methine (CHCl) protons of the PVC backbone, respectively. Hence, it was confirmed that the extracts via Soxhlet extraction were PVC. Furthermore, small peaks at 5.5–6.5 ppm were discovered only after the alkali treatment (Figure 11b), indicating allyl protons [7]. In addition to the AEMs becoming darker by the treatment, C=C double bonds were generated by dehydrochlorination to form a polyene structure along the polymer backbone. Meanwhile, Figure 11c shows the ^13^C NMR spectra of the corresponded specimens. The absence of peaks between 80 and 160 ppm confirmed the absence of other serious degradations that could lead to different chemical structures. In addition to the peaks of the PVC carbon skeleton (44–47 and 55–57 ppm), it was difficult to verify the polyene structure because of the low sensitivity of ^13^C in comparison to those of the protons from the ^1^H NMR spectra.

**Figure 12:**
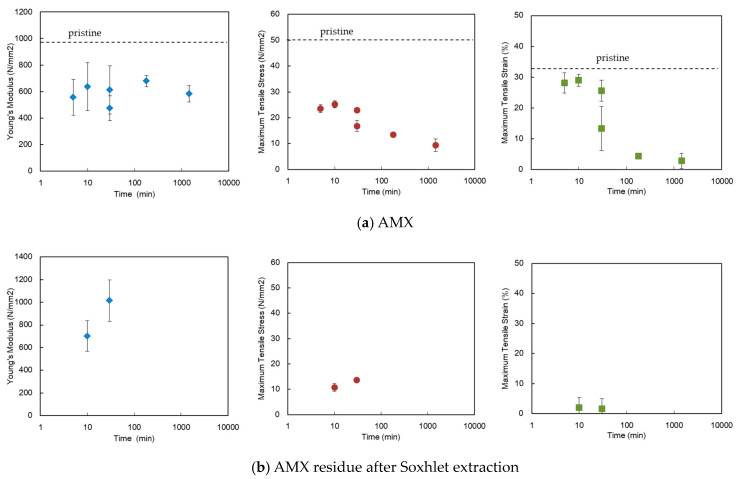
Effect of alkali treatment period on the tensile properties of (**a**) the AMX and (**b**) the AMX residue after Soxhlet extraction.

Furthermore, the effect of alkali treatment time on the mechanical properties of the AEMs was investigated via tensile testing. Figure 12a shows the tensile properties before Soxhlet extraction. The value of the pristine sample was expressed as the dashed line. The tensile stress and tensile strain decreased monotonically as the treatment time progressed. Meanwhile, the properties after the extraction are shown in Figure 12b. The residues extracted from the pristine membrane and following the five-minute treatments were a fibrous powder that could not tolerate the subsequent tensile testing because the PVC skeleton was removed via extraction. In other words, a membrane cannot be formed solely using the CMS-DVB copolymer; however, the shape of the membrane can be maintained using an IPN structure of PVC and CMS-DVB co-polymers. However, the residues after 10 and 30 min of alkali treatment retained their original shape such that tensile testing could be realized. The Young’s modulus and tensile stress increased with treatment time, whereas the tensile strain decreased. The brittle nature is attributable to the cross-linking of the PVC skeleton in alkali conditions, accompanied by the degradation of the polymer. Furthermore, when the alkali treatment exceeded 30 min, the shape of the membrane was maintained; however, it became extremely brittle to withstand the tensile test. This indicates that the main chain breakage has a considerable effect on the mechanical strength, although some of the chains are cross-linked. The mechanism is discussed in detail in the following section.

### 3.4. PVC Deterioration Mechanism

In our previous studies [1,8,9], the dehydrochlorination of the PVC skeleton was confirmed to result in polyene formation under alkaline conditions; furthermore, the formation of a carbonyl group was indicated. In contrast, in this study, the main chain of PVC was partially cleaved and cross-linked. We considered the types of chemical reactions that resulted in these phenomena, as mentioned below. 

**Figure 13:**
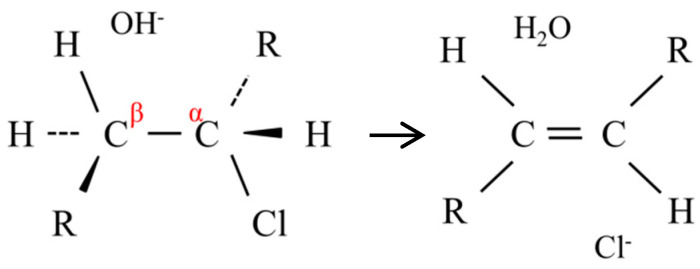
Scheme of the dehydrochlorination.

As shown in Figure 13, OH^−^ first subtracted β-hydrogen on the PVC, thereby inducing dechlorination to form a C=C double bond [10]. 

**Figure 14:**
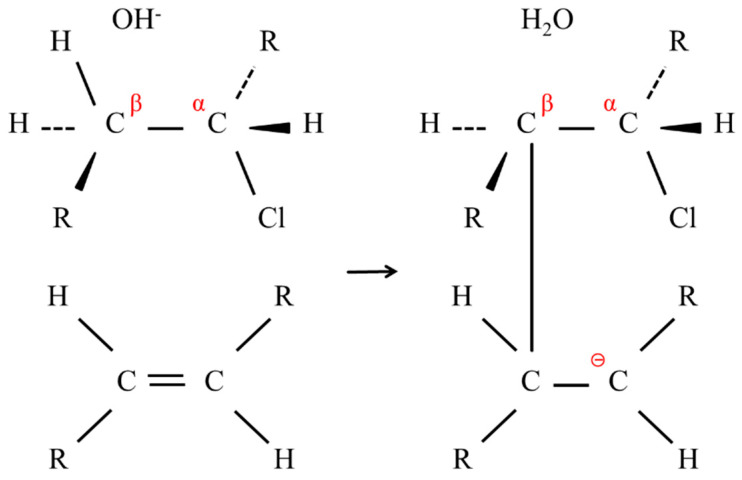
Scheme of forming a cross-linking point.

This typical E2 reaction occurred readily along the polymer backbone, and the conjugation of the C=C double bonds or the polyene formation rendered the membranes darker. In the deprotonation by OH^−^, the resulting carbanion attacked an adjacent C=C double bond to form a cross-linking point, as shown in Figure 14. 

**Figure 15:**
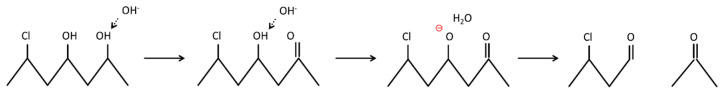
Scheme of the PVC main chain cleaving.

Meanwhile, an SN2 reaction occurred in parallel with the E2 reaction, where the OH^−^ attacked the α-carbons of the PVC in a nucleophilic manner, inducing dechlorination. Subsequently, the OH^−^ further subtracted a proton from the resulting hydroxyl group with the formation of a ketone in the subsequent reaction scheme. When another OH^−^ reacted with a hydroxyl group immediately next to the ketone, the polymer chain was cleaved by a reverse aldol reaction [10]. These processes are shown in Figure 15.

As discussed in a previous study [9], the AMX membrane contains a quaternary ammonium group and can supply OH^−^ continuously to PVC in the AEMs. Meanwhile, CMX contains an anionic sulfonate and prevents OH^−^ from approaching the PVC. This may have caused the deterioration of AMX to be more serious than that of CMX under alkaline conditions.

### 3.5. Deterioration Mechanism

In this study, the burst pressure of the AMX membrane with a PVC backing and binder first increased and then decreased as the alkali treatment time progressed. A similar feature was discovered in the tensile testing. 

**Figure 16:**
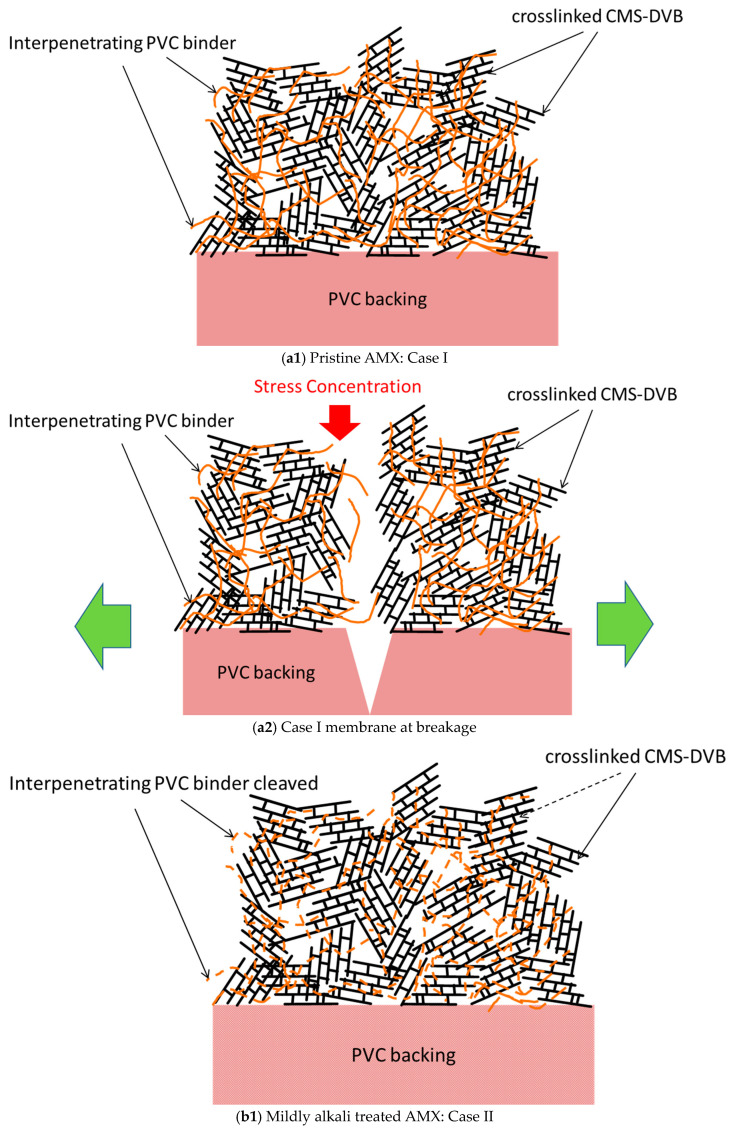

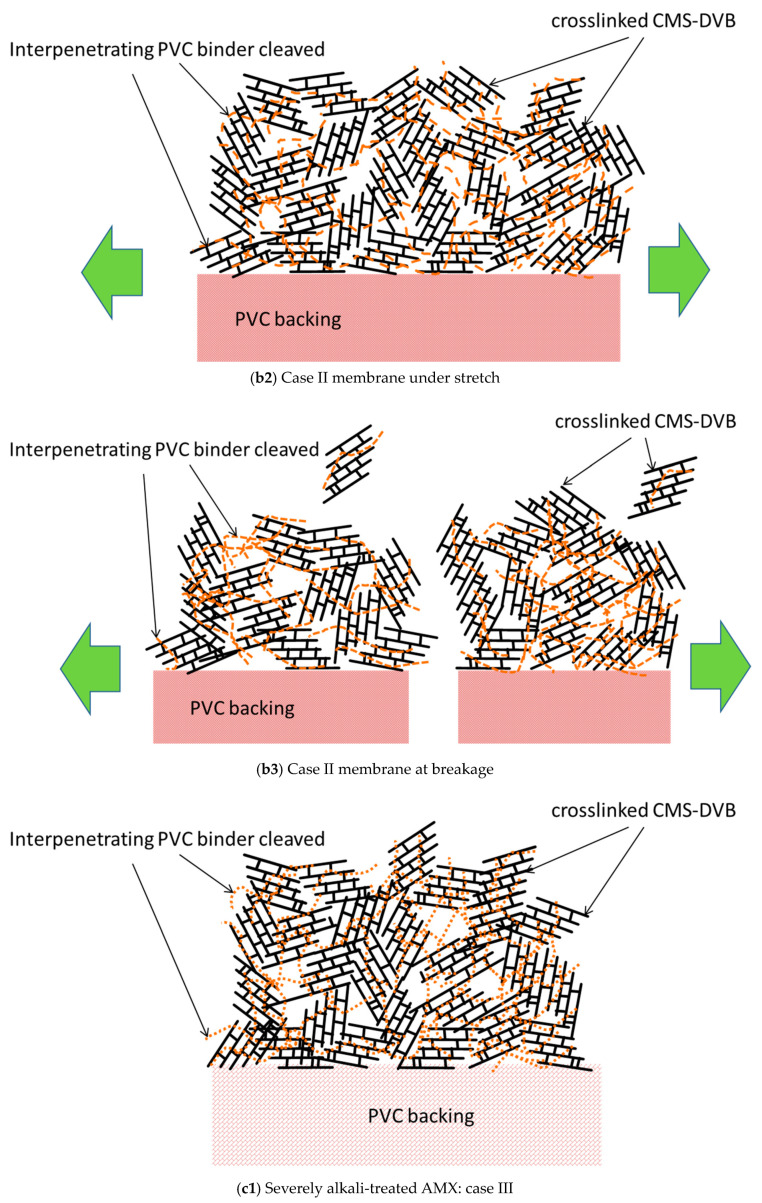

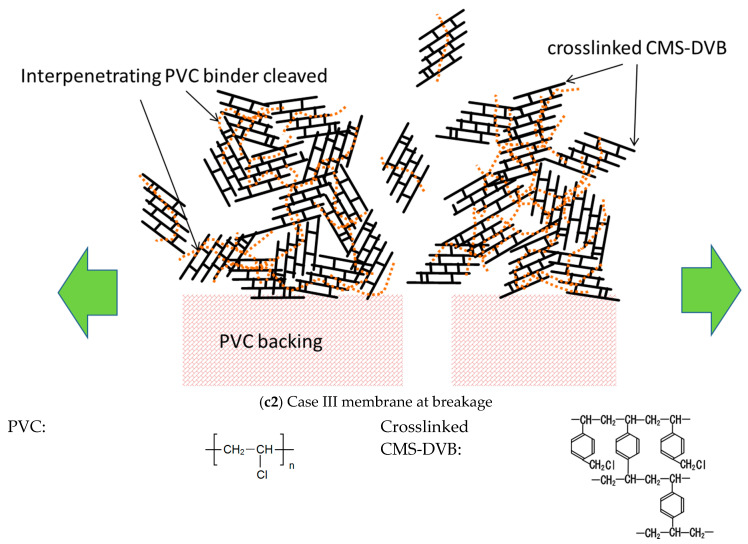
Deterioration mechanism of the AMX membrane. (**a1**) Pristine AMX: Case I; (**a2**) Case I membrane at breakage; (**b1**) Mildly alkali treated AMX: Case II; (**b2**) Case II membrane under stretch; (**b3**) Case II membrane at breakage; (**c1**) Severely alkali-treated AMX: case III; (**c2**) Case III membrane at breakage.

In the previous section, the effect of alkali treatment on PVC was discussed. As indicated, it caused dehydrochlorination with the formation of polyene and cross-linking structures, accompanied by PVC polymer main chain cleavage. We considered this main chain cleavage as contributing to the change in the mechanical properties of the AMX membrane, and proposed the model explaining the deterioration mechanism of the membrane as illustrated in Figure 16.

Figure 16(a1) shows the original structure of the AMX membrane (Case I), which is a composite with a PVC backing and a chloromethylstylene and divinyl benzene (CMS-DVB) cross-linked structure containing a PVC binder (hereinafter, a cross-linked structure). The AMX membrane was prepared via the paste method [1]. A paste containing CMS, DVB, PVC binder, and an initiator was applied to the PVC backing cloth. The PVC binder was compatible with the monomers in the paste. In the thermal polymerization of CMS and DVB in the presence of the binder, the cross-linked structure blocks were randomly formed. This is indicated as the girder bridge structure in Figure 16(a1). The PVC binder was incorporated into the cross-linked structure, resulting in the formation of an interpenetrating network (IPN) structure to interconnect the cross-linked structure blocks, indicated as intertwined chains between the girder bridges. The PVC binder is illustrated in an orange solid line. Hence, the Case I membrane was modeled as a girder bridge cluster connected by PVC chains. In this study, the PVC backing was tough, whereas the cross-linked structure was hard and brittle. When a tensile stress was applied to the Case I membrane, not only the constrained IPN comprising the cross-linked structure blocks and PVC, but also the PVC backing exhibited a high tensile strength, owing to their hardness and brittleness before being fractured without presenting a large elongation, as shown in the SS curve in Figure 3a. The cross-linked structure was fractured first by the stress concentration, followed by the degradation of the PVC backing as shown in Figure 16(a2).

In the Müllen burst test, the fracture of the pristine membrane was accompanied by a loud rupture sound.

The interpenetrating PVC was degraded by the alkali treatment. In the initial stage of degradation, the PVC chain between the cross-linked structure blocks was partially cleaved. It is illustrated in an orange broken line, as shown in Figure 16(b1) (Case II); hence, the girder bridges of the Case II membrane can be transformed more easily than those of the Case I membrane. When stress was applied to the Case II membrane, the girder bridges in the cluster shifted to decrease the stress concentration (Figure 16(b2)), resulting in a yield point and elongation, as shown in the curve in Figure 3b. With further stretching, the resulting partially degraded IPN structure showed multiple yield points until the PVC backing was degraded seriously, as shown in Figure 16(b3). Although the maximum stress at break decreased, the strain to break increased to 189% compared with that of the pristine membrane, and the area below the SS curve increased, resulting in an improvement in the burst pressure. In addition, when the Case II membrane was subjected to a burst test, a “burr” sound was indicated instead of making a cracking sound, suggesting that the backing was torn. The normalized burst pressure increased by 184% compared with that of the pristine membrane.

Not only the PVC chains between the cross-linked structure blocks, but also those in the blocks were cleaved by the further alkali treatment. It is illustrated in an orange dashed line, as shown in Figure 16(c1) (Case III). The severely degraded PVC affected the mechanical properties, as shown in Figure 16(c2), and the resulting membrane exhibited a lower stress at break. The cross-linking of the PVC chain in parallel with the chain degradation would not be negligible during the alkali treatment for a longer period, as it suppressed the elongation under stress. Hence, the strain at break during the tensile testing is as shown in the curve Figure 3c. In addition, the membrane yielded a decrease in the burst pressure by 67% relative to the pristine membrane; hence, the Case III membrane broke without generating a sound.

As described above, this model could explain the improvement in burst pressure and the change in the SS curve of AMX treated with alkali solutions. However, this model should be validated. The experiment conducted in this study should be repeated for a test AEM that uses a PVC backing but does not contain a PVC binder. This will be realized in the near future.

## 4. Conclusions

We systematically investigated the effect of alkali treatment on a commercial AEM containing PVC as a backing and binder and discovered that the alkali treatment improved the burst pressure of the AMX membrane; in some cases, its burst pressure was almost double that of a pristine AMX.

When a tensile test was conducted subsequently, it was discovered that the tensile stress decreased monotonically as the alkali treatment time progressed, whereas the tensile strain improved temporarily, i.e., it became easier to stretch and then decreased. A good correlation was discovered between the burst pressure and the area below the SS curve. It was interpreted that the area below the SS curve corresponded to the work up to the burst.

We conducted a Soxhlet extraction of the immersed membrane and analyzed the chemical structure of the PVC after treatment. The results indicated not only polyene formation, but also main chain cleavage and the formation of a cross-linking structure.

Based on the findings as well as information from an organic chemistry textbook, we interpreted the reaction mechanism and developed a model that elucidated the change in the mechanical strength.

Upon alkali contact, the PVC was dehydrochlorinated by the E2 mechanism and formed a double bond and hence a cross-linked structure. The SN2 mechanism progressed in parallel with the E2 mechanism, and Cl was replaced with OH. The reverse Aldol reaction, which involved oxidization with OH, caused the main chain to break.

The AMX membrane is a composite of “PVC backing” and a “CMS-DVB cross-linked structure containing a PVC binder”. The improvement in the burst strength of the AMX membrane after alkaline treatment is attributed to the cleavage of the main chain of the PVC. Further investigations using a test AEM without a PVC binder are necessary to validate the model.
